# Assessment of two central auditory tests in elderly patients without hearing complaints

**DOI:** 10.1590/S1808-86942011000100005

**Published:** 2015-10-19

**Authors:** Alina Sanches Gonçales, Maria Cristina Lancia Cury

**Affiliations:** 1 MSc in Special Education - UFSCar, PhD student - ENT Department - USP-Campus Ribeirão Preto; 2 PhD in Neurology - Medical School of Ribeirão Preto-USP, Assistant Physician - Hospital das Forças Armadas - Brasília

**Keywords:** hearing, auditory perception, comprehension, aged

## Abstract

Speech understanding disorders in the elderly may be due to peripheral or central auditory dysfunctions. Asymmetry of results in dichotic testing increases with age, and may reflect on a lack of inter-hemisphere transmission and cognitive decline.

**Aim:** To investigate auditory processing of aged people with no hearing complaints.

**Study design:** clinical prospective.

**Materials and Methods:** Twenty-two voluntary individuals, aged between 55 and 75 years, were evaluated. They reported no hearing complaints and had maximal auditory thresholds of 40 dB HL until 4 KHz, 80% of minimal speech recognition scores and peripheral symmetry between the ears. We used two kinds of tests: speech in noise and dichotic alternated dissyllables (SSW). Results were compared between males and females, right and left ears and between age groups.

**Results:** There were no significant differences between genders, in both tests. Their Left ears showed worse results, in the competitive condition of SSW. Individuals aged 65 or older had poorer performances than those aged 55 to 64.

**Conclusion:** Central auditory tests showed worse performance with aging. The employment of a dichotic test in the auditory evaluation setting in the elderly may help in the early identification of degenerative processes, which are common among these patients.

## INTRODUCTION

The increase in life expectancy is responsible for the growth in the elderly population throughout the world and, consequently, it has brought about different health care needs. Health care professionals who follow this population must understand the changes that happen with aging, and hearing is among them. Sensorineural hearing loss is a consequence of aging and it is one of the most prevalent chronic conditions affecting senior citizens, behind only arthritis and hypertension^1^.

Presbycusis is defined as hearing loss associated with aging, arising from the summation of factors which cause physiological degeneration. The changes stemming from aging involve mainly the inner ear and the central auditory pathways. This hearing loss happens to individuals starting in their fifth decades of lives, and the hearing sensitivity changes affect mainly the high frequencies. Structural changes to the auditory nerve and the central auditory pathways are associated with aging and cause important drawbacks in speech recognition^1,^^2^.

The central structures, both the brainstem as well as the brain cortex are affecting the aged population. There can be a general loss of neurons, blood flow reduction, and reduction in glucose metabolism and oxygen consumption^3^.

The prevalence of AP disorders increase significantly with aging. Differences between the ears, non-existent in the young population, increase with age. Such asymmetry can be justified by the progressive deterioration of the corpus callosum because of aging, causing a drop in the inter-hemispheric transfer efficiency^3^.

More recent studies have considered that the age-related drop in cognitive functions, such as working memory, selective attention and information processing speed, have an important impact on the speech of elderly people^4,^^6^.

Thus, speech understanding problems in the elderly population may be caused by peripheral hearing loss, auditory processing (AP) disorders or drop in cognitive skills.

Speech recognition difficulties increase when the signal is distorted, and the most common condition in these cases is the speech signal distortion by another competitive signal. Speech recognition in the presence of noise, dichotic competition and temporal processing are factors which may compromise speech understanding in the elderly^1,^^7^.

There is a greater result variability concerning temporal processing in the elderly, in other words, such difficulty does not affect everyone in the same way^8^.

Because of the large variability in the results found assessing the hearing in elderly patients, numerous authors have carried out studies with the aim of investigating the central hearing in this population, using auditory processing tests.

The effects of aging in the execution of dichotic tests in individuals with symmetrical sensorineural hearing loss were studied in 30 young and elderly individuals. The tests used were the digits, vowel-consonant and the recognition of low redundancy phrases. The results show a worse performance in all these tests among the elderly. There was a poorer performance in the left side hearing when compared to the right side for the same group, showing the effects of chronological age in dichotic hearing tests^4^.

Quintero et al. (2002) compared the performance of elderly individuals with normal hearing ones and those with symmetrical sensorineural hearing loss - characteristic of presbycusis, by means of disyllable recognition test in a dichotic task (SSW). They assessed 100 elderly individuals with ages varying between 60 and 79 years. The SSW test results showed an increase in the mean number of errors for most of the conditions assessed in the study group, and a greater percentage of errors when competitive noise was present, and the competitive left side was the condition with the greatest number of errors. The authors concluded that the sensorineural hearing loss is not a determining factor; however, it is a worsening factor of the difficulty in speech intelligibility in the elderly^9^.

Pinheiro and Pereira, in 2004, carried out a study to characterize the verbal and non-verbal sounds interaction in elderly individuals with and without hearing loss, using Sound Location in Five Directions, Binaural Fusion and Pediatric Sentences Identification (PSI) tests. They assessed 110 normal hearing elderly and those with moderately severe sensorineural hearing loss. The results showed that only the Binaural Fusion test was changed in the population investigated, pointing to losses in the recognition of physically distorted verbal sounds^10^.

A revision study on the masking of language information by competitive speech sounds concluded that elderly adults may have more interference in competitive speech when compared to young adults, with a need for a signal/noise ratio about 2.8dB higher than that for young people in speech recognition in the presence of competitive speech^11^.

A retrospective survey was carried out in order to compare the Speech with Noise test performance of 55 elderly individuals. They analyzed performance in the SRI and Speech with Noise tests. The noise presentation resulted in a negative impact for the elderly without hearing loss. The authors concluded that the performance in the speech test cannot be justified only through their tonal thresholds^12^.

A study with the goal of analyzing the central auditory function efficiency in elderly patients who reported not having hearing difficulties assessed 40 patients with mean age of 68.2 years, without history of neurological disorders. The following tests were carried out: Synthetic Sentences Identification with ipsilateral competitive message (PSI), frequency pattern and SSW. The statistical analysis for the SSW test showed a statistically significant difference between the mean values of the percentage of correct answers under the RC and LC conditions. The authors argued that the changes arising from aging happen to the entire auditory nervous system, and they concluded that the prevalence of central hearing abnormalities show inefficiencies of the auditory function, even in elderly patients who report good hearing^13^.

The interference of different background noises in the speech processing of elderly individuals was studied assessing 24 individuals with symmetrical bilateral sensorineural hearing loss, with ages varying between 56 and 83 years. Word recognition tests in the presence of background noise were employed. The results show that with aging, individuals have lower capability of using the modulated noise intervals to recognize speech^14^.

In an attempt to establish which factors determine faster speech recognition in the elderly, the authors employed compressed speech tests in young and elderly patients alike. The data obtained suggest that information processing problems involve auditory and non-auditory areas. Information processing reduction is part of the problem senior citizens have to sustain speech understanding in situations when the signal is distorted^6^.

One study which investigated speech understanding in the elderly in the presence of competitive noise, by means of functional magnetic resonance, concluded that young adults proved to be more efficient in the hearing cortical areas (temporal cortex, bilaterally) while the elderly have a more diffuse neural network involving frontal and ventral cerebral regions^15^.

The conclusions of the aforementioned studies point to a worsening in the auditory processing of the elderly population, especially regarding speech in the presence of competitive noise and in dichotic hearing tests. They also indicate that there are difficulties in the speech recognition of the elderly, regardless of peripheral hearing loss. Nonetheless, the studies vary considerably in regards of the applied auditory processing test and the degree of sensorineural hearing loss accepted in the sample make up.

With the aim of better understanding the auditory difficulties of the elderly population, this study investigated the auditory performance of elderly patients without auditory complaints in two auditory processing tests.

## MATERIALS AND METHODS

This project was submitted to the Ethics in Research Committee of the institution where it was carried out, and it was approved under protocol # 8952/2004. All participants signed the free and informed consent form.

We assessed 22 volunteers aged between 55 and 75 years, without hearing complaints. Besides the lack of complaints, the other criteria used to include patients in the sample were: no changes seen upon ear inspection; mild sensorineural hearing loss - at most, and symmetrical below 4000Hz; Speech Recognition Index (SRI) greater than 80% and no neurologic disorders or severe chronic diseases.

The patients were submitted to a brief interview in order to confirm the sample inclusion factors, as well as ear inspection and tonal air conduction audiometry in the frequencies of 250 and 8000 Hz. For those patients with air hearing thresholds above 25 dB hearing level (HL), we carried out a bone transmission threshold test in the frequencies of 500 to 4000 Hz. Following that, we investigated the Speech Recognition Threshold (SRT), in order to confirm the air conduction threshold and the speech recognition index (SRI), done at 40 dB sensation level (SL), with the CD from the Central Auditory Processing Assessment Handbook, volume 1 (track 2, D1 and D2 lists) ^16^.

The procedures used for study were the Speech with Noise test and the Alternate Dissyllable Dichotic (SSW) test.

The Speech under Noise test was carried out using the D3 and D4 lists and the track 2 from the aforementioned CD^13^. The monosyllables were presented at 40 dB SL and the *White Noise* was presented ipsilateral in the signal to noise ratio of +5dB. The patients were instructed to repeat the words they heard and ignore the competitive noise.

In order to perform the SSW tests, we used the track 6 from volume 2 of the Central Auditory Processing Assessment Handbook CD^16^. The stimuli presentation intensity was 50 dB SL. The SSW test is made up of 40 sequences of 4 dissyllable words, presented in competitive and non-competitive fashions. The patients were instructed to repeat the sequence of words they heard.

We used the Madsen, Midimate 622 audiometer during the entire assessment.

The results obtained were statistically analyzed by using the SAS® 9.1 software from PROC NLMIXED, in which we used the non-linear regression model with mixed effects. The analysis's goal was to compare genders, ears and age groups in the performance of the auditory processing tests.

For all the calculations, the significance level was set at 5% *(p* value equal to or below 0.05*).*

## RESULTS

Of the 22 individuals evaluated, 12 were females and 10 were males. Their ages varied between 55 and 75 years, and the mean age was 62.82 years, with a standard deviation of 6.23.

The descriptive analysis of the tonal thresholds obtained from the population under study is presented on Table 1. For this analysis, we investigated the 44 ears together, since symmetry between them was considered a sample inclusion criterion.

**Table 1:** Descriptive statistics according to the tonal thresholds obtained from the audiogram.

Frequency (Hz)	Mean	Standard Deviation	Minimum	Median	Maximum
250	14,77	8,07	0,00	15,00	35,00
500	15,34	7,35	0,00	15,00	30,00
1000	13,86	6,09	5,00	10,00	30,00
2000	15,11	8,03	0,00	15,00	30,00
3000	17,39	8,18	5,00	15,00	40,00
4000	21,59	9,51	5,00	20,00	40,00
6000	27,39	12,69	10,00	25,00	60,00
8000	29,32	17,37	5,00	25,00	70,00

Data on Table 1 show minimum thresholds varying between 0 and 10 dB HL for all the ears assessed. Auditory thresholds varied between 30 and 70 dB HL, and up to the frequency of 4,000 Hz the thresholds did not exceed 40 dB HL. The mean and median values were within the thresholds accepted as mild hearing loss for all the frequencies analyzed, characterizing a downward configuration.

Table 2 shows the descriptive analysis of data obtained in the speech recognition index (SRI) distributed per ear.

**Table 2:** Descriptive statistics of the values, in percentage, obtained from the SRI.

EAR	Mean	Standard Deviation	Minimum	Median	Maximum
Right	92,73	3,18	88,00	92,00	100,00
Left	94,73	3,78	84,00	96,00	100,00

Analyzing Table 2, we notice that the minimum values obtained were above 80% and the mean values were higher than 90% of correct answers in both ears. Such result shows that, in ideal hearing situations, all the individuals analyzed have satisfactory performance, which justifies their lack of hearing complaints.

We used as normality criteria for the hearing processing tests, the patterns described in the literature^13,^^17^ for adults, in other words, Speech under Noise tests must have correct answer indexes above 70% and SSW with indexes higher than or equal to 90% in all test conditions.

The descriptive comparison between genders for the Speech under Noise and SSW tests, with the ears grouped is presented on Table 3.

**Table 3:** Descriptive analysis of the results, in percentage of the speech under noise and SSW (competitive and non-competitive conditions) tests, according to gender.

GENDER	TESTS	Mean	Standard Deviation	Minimum	Median	Maximum
	Speech Noise	70,17	7,08	56,00	72,00	84,00
Females	SSW-NC	98,44	1,78	95,00	98,75	100,00
	SSW-C	89,27	8,95	65,00	92,50	100,00
	Speech Noise	74,00	7,28	60,00	72,00	84,00
Males	SSW-NC	99,13	1,47	95,00	100,00	100,00
	SSW-C	94,75	3,13	87,50	95,00	100,00

Legend: SSW = alternate disyllable dichotic test; NC = non-competitive condition; C = competitive condition.

The statistical analysis did not show gender differences for any of the three situations analyzed, in other words, speech with noise *(p = 0.23*), SSW- non-competitive condition *(p = 0.99)* and SSW - competitive condition *(p = 0.18*).

Tables 4 and 5 have the data from the descriptive analysis of the results from the auditory processing tests distributed per ear, being grouped by gender.

**Table 4:** Descriptive analysis of the results in percentage of the SSW test, in the competitive and non-competitive conditions, according to right and left ear.

EARS	Condition	Mean	Standard Deviation	Minimum	Median	Maximum
R	SSW-NC	98,52	1,99	95,00	100,00	100,00
	SSW-C	94,32	4,44	80,00	95,00	100,00
LL	SSW-NC	98,98	1,26	97,50	100,00	100,00
	SSW-C	89,20	8,88	65,00	92,50	97,50

Legend: SSW =alternate disyllable dichotic test; NC = non-competitive condition; C = competitive condition

**Table 5:** Descriptive analysis of the results, in percentage, of the speech-under-noise test, according to right and left ear.

EARS	Mean	Standard Deviation	Minimum	Median	Maximum
Right	70,36	7,05	56,00	72,00	84,00
Left	73,45	7,46	60,00	72,00	84,00

Observing Table 4, we can identify mean indexes higher than 90% of correct answers for both ears in the non-competitive condition and on the right ear for competitive. Only the left competitive ear had mean values below 90% of correct answers. Moreover, we can notice that only in the left competitive condition no individual reached 100% of correct answers and it was this condition which had the minimum percentage of the lowest correct answers.

On Table 5, we can see correct answers mean values higher than 70% in the speech under noise test for both ears.

The application of the comparative tests showed a statistical difference for the ears only in the competitive condition for the SSW test, and *p value* < *0.01*. The comparison between the right and left ears for the speech under noise test had p *= 0.25* and for the SSW non-competitive, p= 0.39.

Considering the degenerative process arising from aging, which impairs performance in dichotic hearing tasks, and in the fact that the population studied had age distribution involving two decades of life, we chose do carry out statistical analysis of this population distributed in two age groups, in other words, one group with age varying between 55 and 64 years, called Group 1; and another group with ages between 65 and 75 years, called Group 2.

Following, we present the results from the statistical analysis of the same population, with a new distribution. Group 1 (G1), made up of 14 individuals with mean age of 58.64 years; and group 2 (G2), with 8 individuals with mean age of 70.12 years.

The SSW test descriptive analysis in the competitive and non-competitive conditions according to groups G1 and G2 and right and left ears are depicted on Table 6.

**Table 6:** Descriptive statistics of the results, in percentage, of the SSW test, according to ear, distributed in the two age groups of the study.

Age Group	Ear	Condition	Mean	Standard Deviation	Minimum	Median	Maximum
G1	Right	SSW-NC	98,75	1,90	95,00	100,00	100,00
		SSW-C	95,36	3,38	90,00	96,25	100,00
	Left	SSW-NC	99,29	1,17	97,50	100,00	100,00
		SSW-C	92,32	5,14	77,50	93,75	97,50
G2	Right	SSW-NC	98,13	2,22	95,00	98,75	100,00
		SSW-C	92,50	5,67	80,00	93,75	97,50
	Left	SSW-NC	98,44	1,29	97,50	97,50	100,00
		SSW-C	83,75	11,57	65,00	86,25	97,50

Legend: SSW = alternate disyllable dichotic test; NC = non-competitive condition; C = competitive condition

**Figure 1:**
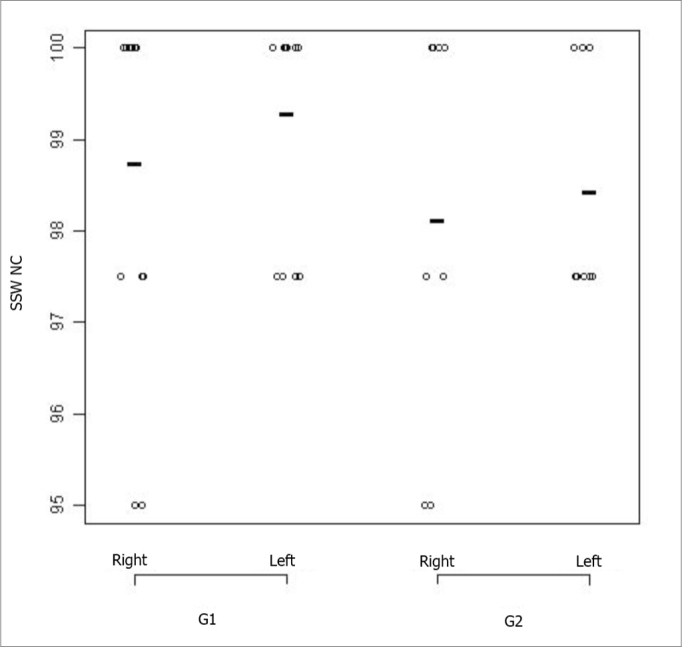
SSW test results plot - non-competitive condition, according to age range and ear. - SSW-NC: alternate disyllable dichotic test - non-competitive condition; G1: group 1; G2: group 2

**Figure 2:**
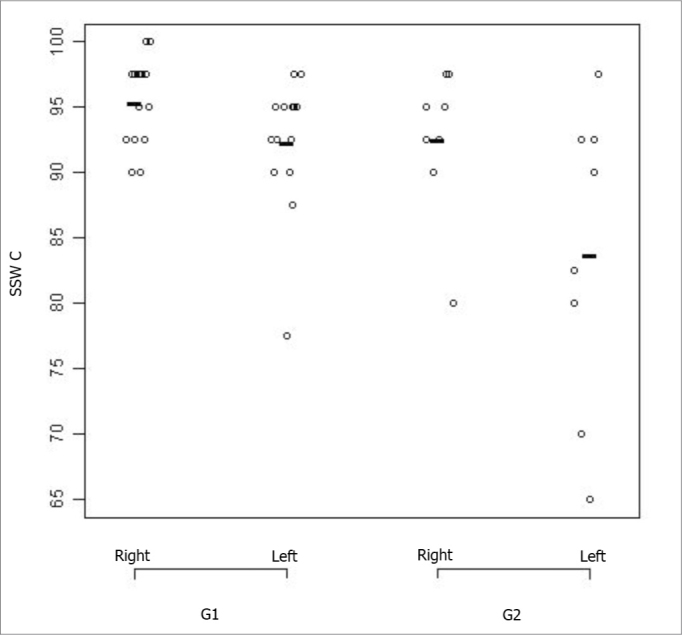
SSW test results plot -competitive condition, according to age range and ear - SSW C: alternate disyllable dichotic test - competitive condition; G1: group 1; G2: group2

Figures 1 and 2 correspond to the graphical distribution of these variables according to age group and ears. We notice that the results from both ears are similar for the non-competitive condition in both age groups, with minimum, medium and maximum correct answers indexes higher than 90%. As to the competitive conditions, we noticed that the left ear is the one with the highest variability of results.

Also in Figure 2, we notice that in the G1, the left ear showed worse results than the right ear. The figure also shows that the right ear in G2 is similar to the left ear in G1. And, finally, the left ear in G2 had the worst performance in the SSW test under the competitive condition.

The comparative analysis did not show statistically significant difference between the groups in the non-competitive conditions for the SSW test. Nonetheless, for the competitive condition we obtained a statistical difference between the groups (p value *0.01)*, which leads us to conclude that G2 (elderly) had the worst results.

**Figure 3:**
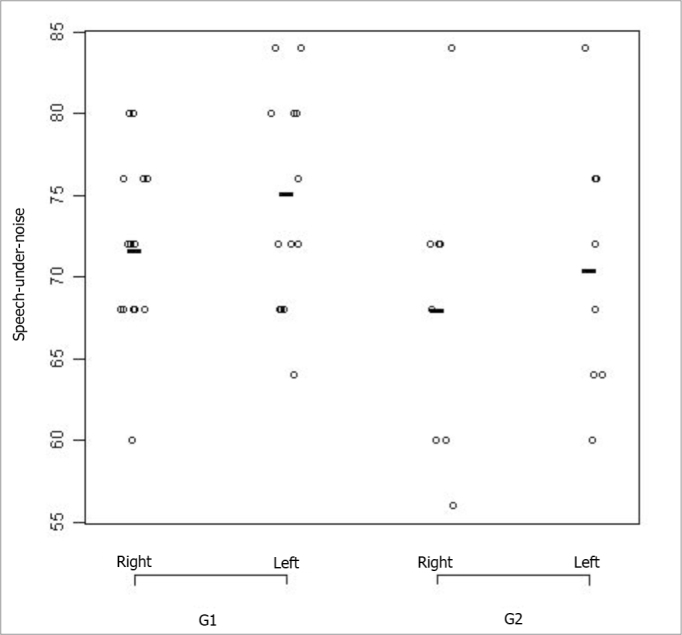
Speech-under-noise test results plot according to age range and ear. - G1: group 1; G2: group 2

In the comparison between the right and left ears from each group, we found a statistically significant difference only for G2 (*p= 0.02).* The statistical comparison of G1 ears, seen on Figure 3, did not show significant differences *(p = 0.09).*

The comparison of the same ear between groups showed a statistically significant difference for the left ear *(p = 0.01)*, once again pointing to better results in G1, when compared to G2.

Table 7 shows the descriptive analysis of the speech under noise test according to groups G1 and G2 and left and right ears.

**Table 7:** Descriptive statistics of the results, in percentage of the speech-under-noise test, distributed in the two age groups of the study.

Age Group	Ear	Mean	Standard Deviation	Minimum	Median	Maximum
G1	Right	71,71	5,54	60,00	72,00	80,00
	Left	75,14	6,87	64,00	74,00	84,00
G2	Right	68,00	9,07	56,00	70,00	84,00
	Left	70,50	7,98	60,00	70,00	84,00

Figure 3 corresponds to the graphical distribution of these variables according to age group and ears. In it, as well as in Table 7, we can see that the results are slightly better for Group 1, in other words, in the group of individuals in the age range between 55 and 64 years.

The comparison between age groups showed a statistical difference for the speech under noise test *(p < 0.01).* When we compared the right and left ears within each group, we found a statistically significant difference for G2 only *(p = 0.01).* When we compared the same ear between the groups we found a statistically significant difference *(p < 0.01)*, in which the right ear performance was worse for the higher age group.

## DISCUSSION

As of the fifth decade of life, people begin to suffer the effects of aging on the central and peripheral auditory systems. Nonetheless, the differences arising from the body and/or the communication needs of each individual, make these progressive difficulties to be perceived very differently by people^1^^-^^2^. It is likely that this is the justification for elderly people reporting good hearing, even after hearing loss onset.

To try to find people older than 60 years of age with hearing thresholds below 25 dB HL is not an easy task. Many who came and denied hearing and oral understanding differences had a descending-type hearing loss, even initially. Thus, it was very difficult to find a group of people in this age range without an air conduction threshold higher than 25 dB HL. Such fact made us abandon this initial criterion of sample composition and made us accept people with thresholds up to 40 dB HL in frequencies of 500 to 4,000Hz, as long as they did not report hearing difficulties.

In our study, of the 22 individuals assessed, only 9 (40%) had thresholds higher than 25dB HL in all the frequencies bilaterally, 6 (27%) of them were below 65 years and only 3 (13%) were older than 65 years.

Other studies involved elderly individuals without auditory complaints to make up the sample and also found the same difficulty. Sanchez et al. (2008), studied 40 elderly individuals and found only 20% of them with thresholds within normal parameters for all the frequencies^13^. Pinheiro and Pereira (2004), in a population of 110 elderly, found 91 (83%) individuals with hearing loss^10^.

Quintero et al. (2002) found 50 people in the age range between 60 and 79 years with thresholds in all the frequencies of the audiogram lower than or equal to 5 dB HL. This study concluded that the sensorineural hearing loss cannot be considered a determining factor of the difficulties in speech intelligibility in the elderly in noisy or reverberating environments^9^.

The speech recognition index (SRI) above 80% in all the participants in the study justifies the absence of complaints as to speech inteligibility^10^.

Of all that has been said, we notice that peripheral hearing loss, even gradual, is not perceivable, since it starts in the elderly population and is in agreement with the extensive literature on the topic^1^^-^^2,^^8^, ^9^, ^10^.

Knowing that the prevalence of auditory processing disorders grows with aging^7^, we can assume a certain difficulty in central auditory skills, of slow and progressive onset, which can take a while to be perceived by the elderly population.

As far as gender is concerned, we did not find performance differences for the tests studied. Other studies involving speech-under-noise tests^12^, synthetic phrases identification (SSI) tests with ipsilateral competitive message, frequency pattern test and alternate dissyllable dichotic test^13^ also did not find influence of gender in the results found.

Insofar as the differences between ears are concerned, we found asymmetry, in other words, a worse performance for the left ear only in the SSW competitive condition.

The interaural differences increase with age and can be justified by the structural and cognitive models^5^. The two models try to explain the right ear advantage and consequent left ear disadvantage in the dichotic tests. This asymmetry happens, partially, because of a drop in cognitive skills and partially because of a drop in the efficiency of transferring information from one brain hemisphere to the other.

The structural model proposed by Kimura (1961), justifies the perception asymmetry as follows: the information presented to the right ear travels directly to the left hemisphere. During dichotic stimulation, the ipsilateral auditory pathways are suppressed favoring the contralateral ones, which have a greater number of fibers. The left ear disadvantage is a product of the greater verbal information transmission time in this ear, since it has to be moved from the right hemisphere for its processing in the left hemisphere, through the corpus callosum. Therefore, the left ear requires greater corpus callosum participation in order to be efficient in language information processing. In the case of the elderly, this central nervous system structure suffers the natural deterioration of aging and its performance drops, causing the ear asymmetry hereby observed^18^^-^^19^.

The cognitive model emphasizes the importance of attention, working memory and information processing speed in the dichotic hearing tasks. Because of the left hemispheric dominance for speech processing, most people have dominance as to the stimuli heard on the right side, which allows them to predominantly use a more automatic acoustic processing of the stimuli (*bottom-up*). On left-side hearing (dichotic task), the stimuli are naturally suppressed by stimuli in the right ear. In order to serve the need for directing listening to the left, it is necessary to have a greater activation and involvement of the cognitive function (*top-down*). Since these functions deteriorate with aging, the ear asymmetry can be seen during dichotic tests given to the elderly^4^^-^^5^.

None of the two models is able to justify alone the effects of aging in the asymmetry of the ears, therefore, there can be an association of both in the dichotic hearing situations^5^.

Although the studies vary as to the dichotic tests employed, the task required, as well as the participating populations, there is one common point to these studies: the constant finding of ear asymmetry with a worse left ear performance in dichotic tasks involving integration, as well as in listening guiding tasks^4,^^9,^^13,^^20^.

There are still papers which show important evidence of left ear worse performance in cases of degenerative disorders, such as Alzheimer disease^19,^^21^.

Based on what was mentioned above, our finding of asymmetry in the SSW test (competitive condition) seems to corroborate what is published in the literature, and it can be considered a reflex of aging.

The separation of this same population in two age groups brought us new evidence that the advancement of age makes the sound signal processing deteriorate, since the comparisons between the younger group (G1) and the elderly (G2) have statistically significant differences in the two tests investigated, both in the monotic as in the dichotic tasks. Such findings bring us back to the existence of neuronal losses which are common to aging^8^.

In the SSW non-competitive test, a situation in which one hears a word alone, without any type of sound competition, the performance from the participants of the two groups was similar and with correct answers indexes close to those from young adults^17^.

The aforementioned information makes us consider that: age-related changes happen to the entire central nervous auditory system; the prevalence of auditory changes show the inefficiency of the central auditory functions even in elderly people without complaints^13^; the difficulties the elderly have in processing information may stem not only from the auditory system, but also from the cognitive deterioration inherent to aging^4,^^5,^^15^; and the peripheral hearing is not the determining factor in these difficulties^5,^^9,^^12^, since the assessed groups showed a hearing loss symmetrical with the same characteristics.

The last discussion to which the data from this study refer to is the worse right ear performance in the speech test with noise in both groups (G1 and G2). The literature states that there is a worsening in the speech intelligibility of the elderly when exposed to noisy environments^1,^^7^. Studies have shown that elderly persons need a signal/ noise ratio higher than young individuals for speech understanding^11^, than the background noise interference on the understanding task depends on the type of task and the degree of redundancy of the speech material^14^, and that the elderly have a more diffuse neural network involving frontal and ventral regions during speech understanding under noise^15^. Nevertheless, no analyzed study involved the different performance of the ears in elderly individuals concerning speech intelligibility test in the presence of noise. In the Central Auditory Processing Assessment Handbook, there is a reference to the worse performance in the first ear tested in relation to the second one^22^. Since all the individuals in our sample did the test first in the right ear and later on the left ear, there is the likelihood that this result may have suffered the interference of the procedure adopted for data collection and not representing exactly an aging-related difference. Later studies may be useful to analyze this variable more in depth.

## CONCLUSION

Age caused a worsening in the performance of the elderly in the Speech-under-noise and alternate disyllable dichotic (SSW) tests, regardless of a hearing complaint.

The left ear has a worse performance when compared to the right one in dichotic tests, which can be associated to central auditory pathway and cognitive functions deterioration associated with aging.

The introduction of the dichotic test to the audiological battery of tests in the elderly with and without peripheral auditory loss may contribute to the identification of the degenerative process and, consequently, for the creation of intervention strategies before they interfere in the communication skills of these individuals.
